# The long-term survival in primary retroperitoneal mucinous cystadenocarcinoma: a case report

**DOI:** 10.1186/s40792-017-0394-z

**Published:** 2017-11-25

**Authors:** Hirotaka Tokai, Yasuhiro Nagata, Ken Taniguchi, Naomi Matsumura, Amane Kitasato, Takayuki Tokunaga, Hiroaki Takeshita, Tamotsu Kuroki, Shigeto Maeda, Masahiro Ito, Hikaru Fujioka

**Affiliations:** 1 Department of Surgery, National Hospital Organization Nagasaki Medical Center, Kubara 2-1001-1, Omura, Nagasaki, 856-8562 Japan; 2 Department of pathology, National Hospital Organization Nagasaki Medical Center, Kubara 2-1001-1, Omura, Nagasaki, 856-8562 Japan; 3 Center for Comprehensive Community Care Education Nagasaki University Graduate School of Biomedical Sciences, Nagasaki, Japan

**Keywords:** Retroperitoneal, Cystadenocarcinoma, Mucinous

## Abstract

**Background:**

Primary retroperitoneal mucinous cystadenocarcinoma (PRMC) is extremely rare, and its biological behavior, pathogenesis, optimum treatments, and prognosis remain to be elucidated. We herein report a case of PRMC with an 80-month follow-up.

**Case presentation:**

A 29-year-old woman was diagnosed with unknown retroperitoneal tumor with benign right ovarian cyst and uterine fibroids, and she underwent laparotomy. The tumor was completely resected with a subsequent histopathological diagnosis of primary retroperitoneal mucinous cystadenocarcinoma (PRMC). Eighty months after surgery, she remains recurrence-free.

**Conclusion:**

PRMC is an extremely rare tumor. Only around 60 cases have so far been published in the literature. The preoperative diagnosis of PRMC is difficult, and a definitive diagnosis can usually only be made based on the findings of histopathological examinations after surgery. Presently, only radical resection is useful for both diagnostic and therapeutic purposes. The optimal long-term management after surgery is still not well established. Further studies on PRMC are therefore needed to elucidate the etiology and establish effective treatments.

## Background

Primary retroperitoneal mucinous cystadenocarcinoma (PRMC) is extremely rare, with the first case was reported in 1965 [1]. Since then, only 61 cases have been reported in the English literature, to our knowledge. Little is known about the biological behavior and pathogenesis of this disease. The diagnosis is often confusing preoperatively. In addition, the optimum treatments and prognosis of PRMCs remain to be uncertain. We herein report a case of PRMC with 80 months of follow-up.

## Case presentation

**Fig. 1:**
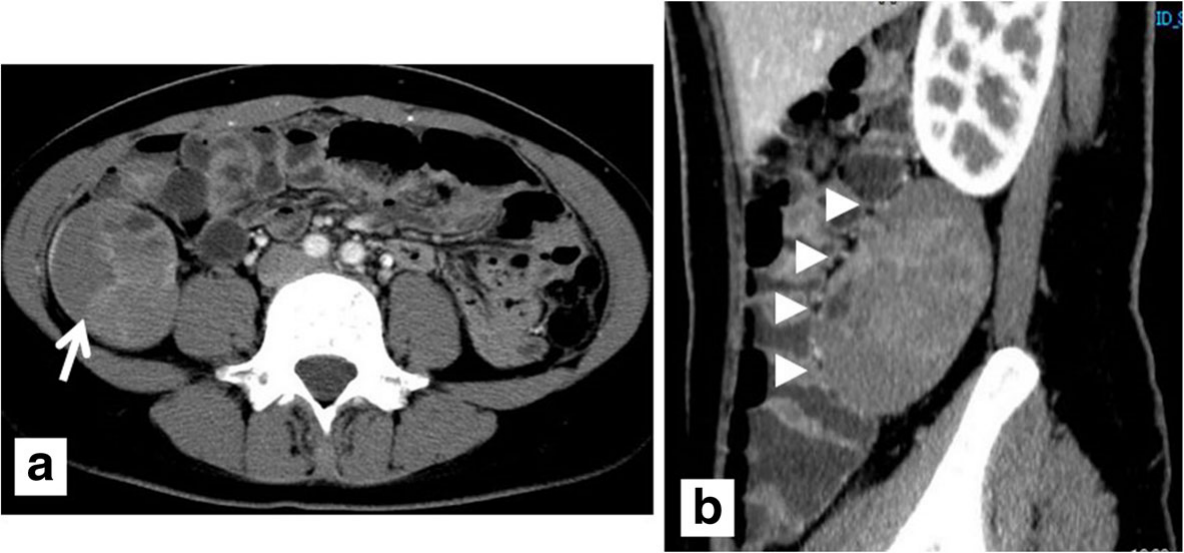
(**a**) Horizontal and (**b**) sagittal image of CT reveals a cystic and well-defined tumor 8.5 cm in diameter behind the ascending colon (arrow heads) with enhanced and segmented components (arrow)

**Fig. 2:**
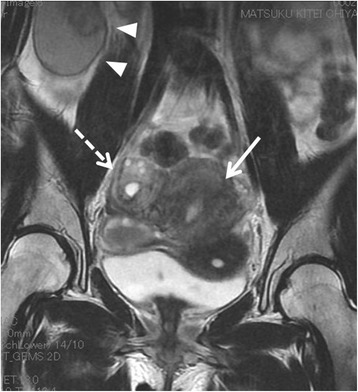
MRI demonstrates a cystic mass of iso-intensity on T1-weighted and high intensity on T2-weighted images (arrow heads), uterine fibroids (arrow), and cystic masses at the right ovary (broken arrow)

A 29-year-old woman was admitted to our hospital for further examination due to abdominal pain and a cystic mass in the right lower abdomen. A physical examination revealed no tumor in her abdomen on palpation. Contrast-enhanced computed tomography (CT) revealed a cystic and well-defined tumor of 8.5 cm in diameter behind the ascending colon. The tumor had enhanced and segmented components (Fig. 1). Magnetic resonance imaging (MRI) demonstrated a mass behind the ascending colon of iso-intensity on T1-weighted and high intensity on T2-weighted images. Other bi-cystic masses of 8 cm in diameter at the right ovary and uterine fibroids were also observed (Fig. 2). There was no evidence of invasion to adjacent tissues or distant metastasis. Based on imaging findings, the differential diagnosis of this tumor was thought to be neurogenic tumor, leiomyosarcoma, malignant fibrous histiocytoma, and primary retroperitoneal tumor. The serum levels of carcinoembryonic antigen (CEA) were within normal limits, while those of cancer antigen (CA) 19-9 and 125 (CA125) were elevated. She was diagnosed with unknown retroperitoneal tumor with benign right ovarian cyst and uterine fibroids.

Laparotomy was performed, revealing a tumor capsulated with a thin wall behind the ascending colon, an ovarian cyst, and uterine fibroids. The tumor was completely removed without injury of its capsule. The ovarian cyst was enucleated, but the uterine fibroids were not removed in accordance with the preoperative informed consent.

**Fig. 3:**
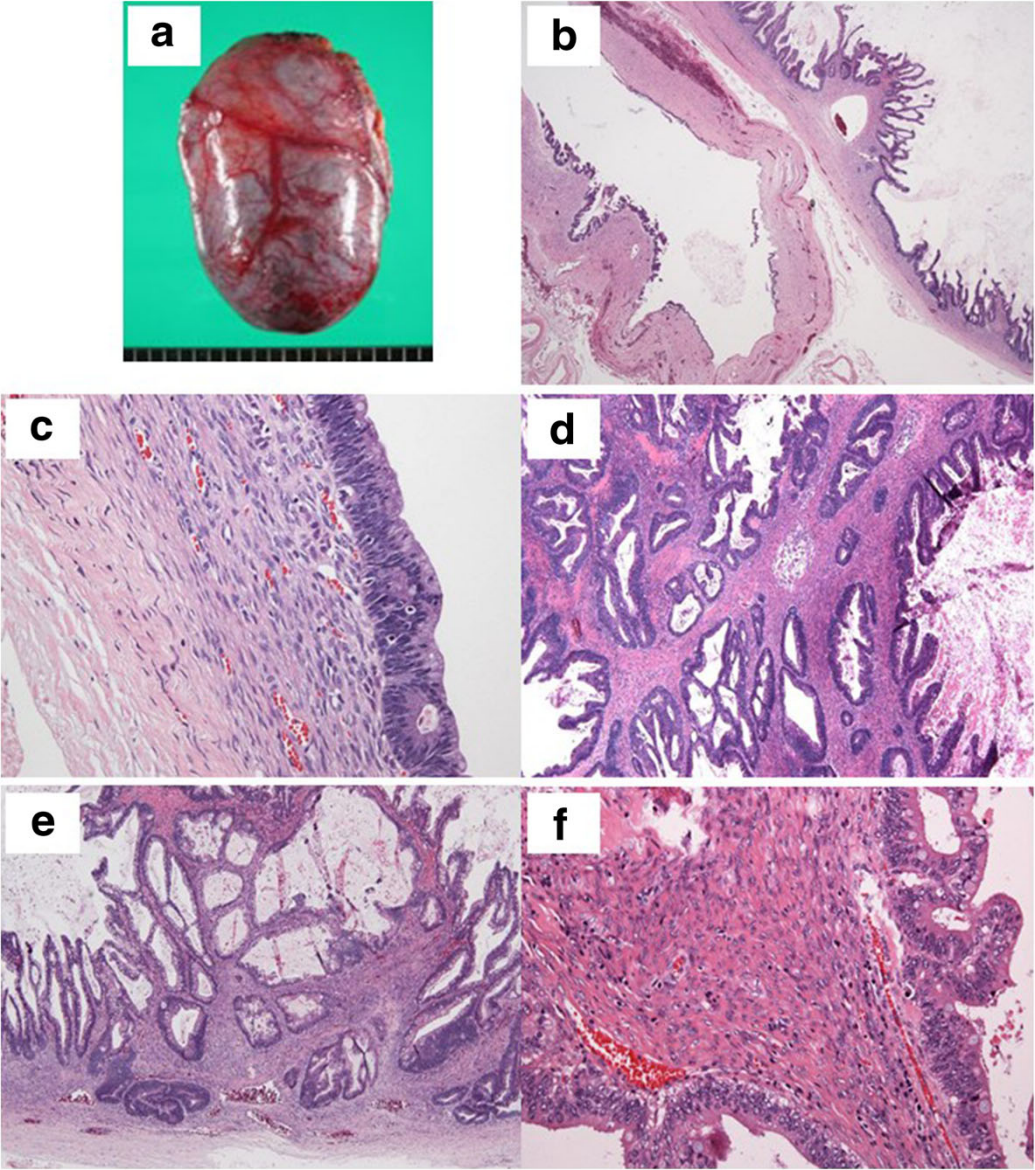
Gross appearance of the specimen (**a**). Microscopic images show that the tumor consists of a unilocular cyst and a large mural solid nodule (**b**). In the nodule, multi-cystic lesions covered by papillovillous hyperchromatic cells are observed. They comprise intestinal-type epithelium with goblet cells (**c**). Some of them are invasive adenocarcinoma cells (**d**) with stromal invasion (**e**). Ovarian-like stroma is also observed (**f**)

**Fig. 4:**
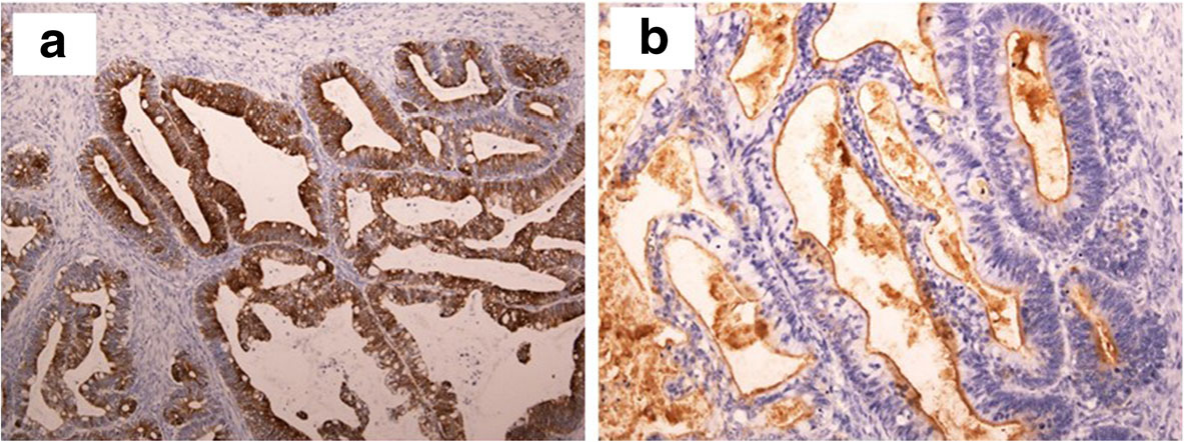
An immuno-histopathological examination revealed positivity for CK 7 (**a**) and CEA (**b**) but negativity for CK 20 in the tumor cells

A histopathological examination showed that the tumor was unilocular with a large mural solid nodule. In the nodule, there were multi-cystic lesions covered with papillovillous hyperchromatic cells made of intestinal type epithelium ranging from adenoma- to invasive adenocarcinoma type. In addition, ovarian-like stroma was seen in the cyst wall, but ovarian tissue and teratomatous elements were not (Fig. 3). An immuno-histopathological examination revealed positivity for CK 7 and CEA but negativity for CK 20 in the tumor cells (Fig. 4), and negativity for estrogen and progesterone receptors in the ovarian-like stromal cells. Considering the histopathological features and the location, the tumor was diagnosed as PRMC without sarcomatous change.

The surgical margin was free from tumor cells. The right ovarian cyst was diagnosed as an endometrial cyst (chocolate cyst). After the surgery, she had no major complications and left our hospital 8 days later. During the follow-up period of 80 months, neither local recurrence nor distant metastasis was found. Her other ovarian cysts, however, gradually enlarged, so she underwent surgery to remove them 4 years after the initial surgery. A histopathological examination revealed that they were benign chocolate cysts. The serum levels of CA 125 and CA 19-9, which had been elevated before the second surgery, normalized after surgery.

### Discussion

Retroperitoneal mucinous cystic neoplasms, including cystadenoma and cystadenocarcinoma, are so rare that the accurate incidence is not available. We searched the PubMed database for published English studies using the terms “primary” and “retroperitoneal” and “mucinous” and “cystadenocarcinoma” or “adenocarcinoma.” Cases of adenomas, borderline tumors, and metastasis to the retroperitoneum were excluded, but mixed-type tumors were included. Our present case is only the 62nd case (Table 1) [1–46]. The mean age is 44.7 years (range 18–86 years), and the mean tumor size is 13.6 cm (range 3 to 26 cm). Only five cases were reported in males. To our knowledge, among the 51 cases in which the follow-up period was mentioned (range 1–130 months; median 16 months), only 7 cases had been followed for over 5 years. They are all alive without recurrence of disease. Although most PRMCs tend not to develop further disease, some with sarcoma-like or anaplastic components have a very aggressive character and can metastasize. Myriokefalitaki [9] reported that the 5-year overall survival was 75.4%.

**Table 1:** Published English studies searched in PubMed

Case no.	Author	Sex	Age	Diameter(cm)	Adjuvant chemotherapy	Follow-up (month)	Status
1	Douglas 1965 [1]	F	18	5	No	N/R	DOD
2	Tykkä 1975 [2]	F	23	10	No	11	DOD
3	Roth 1977 [3]	F	48	N/R	No	6	DOD
4	Fujii 1986 [4]	F	69	23	No	36	NED
5	Nelson 1988 [5]	F	35	20	No	22	NED
6	Chida 1990 [6]	F	42	N/R	No	N/R	N/R
7	Seki 1990 [7]	F	42	11	No	N/R	N/R
8	Park 1990 [8]	F	40	24	Yes	3	NED
9	Jorgensen 1991 [9]	F	38	8	No	9	NED
10	Søndergaard 1991 [10]	F	37	13	No	18	NED
11	Gotoh 1992 [11]	F	44	12.5	Yes	4	DOD
12	Tenti 1994 [12]	F	46	20	Yes	33	NED
13		F	45	20	No	19	NED
14	Motoyama 1994 [13]	F	42	11	N/R	N/R	N/R
15	Carabias 1995 [14]	F	43	15	No	24	NED
16	Lee 1996 [15]	F	55	20	No	30	NED
17		F	45	17	No	15	NED
18	Dore 1996 [16]	F	45	20	No	16	NED
19	Uematsu 2000 [17]	F	86	23	No	72	NED
20	Suzuki 2001 [18]	F	40	15	No	15	NED
21	Tangjitgamol 2002 [19]	F	41	12	Yes	18	NED
22	Kessler 2002 [20]	F	38	11.5	N/R	60	NED
23	Mikami 2003 [21]	F	38	16	Yes	18	DOD
24	Song 2005 [22]	F	72	12	No	4	DOD
25	Sonntag 2005 [23]	F	60	5	No	12	NED
26	Thamboo 2006 [24]	M	64	24	No	18	NED
27	Fan 2006 [25]	F	68	17	No	N/R	N/R
28	Law 2006 [26]	F	35	11	No	60	NED
29	de Leon 2007 [27]	F	36	19	Yes	8	AWD
30		F	21	26	No	6	NED
31	Kashima 2007 [28]	F	28	17	No	13	NED
32	Lee 2007 [29]	F	32	15	Yes	42	NED
33	Green 2007 [30]	M	83	26	No	6	NED
34	Tjalma 2007 [31]	F	74	3	Yes	31	DOD
35	Moral 2008 [32]	F	47	24	No	8	NED
36	Youssef 2008 [33]	F	70	10	Yes	24	NED
37	Roma 2009 [34]	F	35	13	No	13	NED
38		F	47	21	No	1	NED
39		F	24	18	No	2	NED
40		F	43	10	No	5	DOD
41		F	40	11	No	9	DOD
42		F	27	8	No	11	NED
43		F	63	7.5	No	14	AWD
44		F	31	18	No	26	AWD
45		F	48	26	No	58	AWD
46		F	40	15	No	58	NED
47		F	35	N/R	No	91	NED
48		F	49	11	No	130	NED
49		F	20	N/R	No	N/R	N/R
50	Hrora 2009 [35]	M	42	5	No	6	NED
51	Dierickx 2010 [36]	F	50	13	Yes	58	NED
52	Jian 2011 [37]	F	21	14.6	Yes	6	AWD
53	Kanayama 2012 [38]	F	40	25	No	6	AWD
54	Feng 2013 [39]	M	63	4	No	13	NED
55	Hanhan 2014 [40]	F	37	22	No	N/R	N/R
56	Shiau 2013 [41]	M	59	7.5	No	79	NED
57	Kurita 2014 [42]	F	30	19	No	32	AWD
58	Kamiyama 2015 [43]	F	62	10	No	15	DOD
59	Cupp 2015 [44]	F	39	20	Yes	N/R	N/R
60	Dong 2014 [45]	F	52	3.8	No	N/R	N/R
61	Myriokefalitaki 2016 [46]	F	56	24	No	17	NED
62	Present case	F	28	8.5	No	80	NED

*N/R* not recorded, *DOD* dead of disease, *NED* no evidence of disease, *AWD* alive with disease

The etiology and biological behavior of PRMCs are still unclear; however, some hypotheses have been proposed to explain the genesis of these tumors as follows: (1) heterotopic ovarian tissue [3, 11, 47], (2) monodermal variant of teratomas [22, 48], (3) intestinal duplication [49], and (4) coelomic metaplasia [4, 8, 12, 50]. In our case, ovarian-like stroma was histopathologically found in the tumor, although no definitive evidence of ovarian tissue was observed, which was also supported by the results of an immunohistochemical examination of the estrogen and progesterone receptors. These findings exclude the hypothesis of heterotopic ovarian tissue. In addition, the hypotheses of teratoma and intestinal duplication can also be excluded because of the lack of structures of teratoma or well-developed intestinal mucosa and smooth muscle. The fourth hypothesis, which is most well-described in the previous literature, is that PMRCs occur from invaginations of the peritoneal epithelium during embryogenesis. Those invaginated coelomic epithelial cells form cysts that may act like epithelial ovarian tissue and undergo the process of Müllerian differentiation. Eventually, the coelomic epithelia of these cysts undergo metaplasia and develop a spectrum of histological cells in different stages. In our case, the ovarian-like stroma and intestinal type epithelium ranged from adenoma-type mucinous cells to invasive adenocarcinoma cells, which may be explained by the last theory. In addition, the pattern of immunohistochemical expression of CK 7 and CK 20 is similar to those seen in ovarian and pancreatic mucinous neoplasms.

There are no pathognomonic clinical or radiological findings for PRMC, making the preoperative diagnosis of this disease challenging. PRMCs usually present as a multi- or unilocular cystic mass, varying in size and localized anywhere in the retroperitoneal space. Regarding the imaging findings, the diagnostic value of computed tomography and MRI is similar, but MRI can further characterize these lesions and identify their mucinous component. Notable radiographic findings may include thickening and calcification of the cyst wall or mural nodules on imaging that may suggest malignant lesions [27]. Aspiration cytology and/or a biopsy may help with the diagnosis, although they carry risks of recurrence and dissemination in cystic tumors. Serological investigations provide limited diagnostic utility. Tumor markers, including CEA, CA 125, and CA 19-9, are also not very helpful for differentiating from other benign tumors, including ovarian cyst, cystic lymphangioma, and cystic methothelioma. Indeed, in our case, the elevation of serum levels of CA19-9 and CA 125 was also observed during the follow-up period. However, these levels normalized after she underwent a second surgery to resect the benign ovarian cysts. Therefore, imaging examinations such as CT or ultrasonography are the most effective tools for performing follow-up for PRMC.

The management of PRMC is not well established. There is currently no significant chemotherapy for PRMC. The commonly used chemotherapeutic regimes were cyclophosmide and adriamycin, cyclophosmide, adriamycin and cisplatin, cisplatin alone, carboplatin and paclitaxel, or carboplatin alone. Of the 12 patients who received adjuvant chemotherapy, 5 had recurrence (41.7%). Therefore, radical tumor excision is clearly mandatory. Radical resection without rupture is the standard therapy and the most important prognostic tool [46], but whether lymphadenectomy or adjuvant chemotherapy provide benefit is still controversial [11, 12, 19, 21, 27, 29, 31, 36].

## Conclusions

PRMC is a rare tumor that can have an aggressive potential for recurrence. The diagnosis remains difficult preoperatively, and surgeons should be aware of this disease as a differential diagnosis of large retroperitoneal cystic masses with indolent symptoms. The long-term management after surgery is not well established yet. Further studies about PRMC are needed to elucidate the etiology and effective treatments.

## Abbreviations

CA: Cancer antigen
CEA: Carcinoembryonic antigen
CT: Computed tomography
MRI: Magnetic resonance imaging
PRMC: Primary retroperitoneal mucinous cystadenocarcinoma

## References

[1] Douglas GW, Kastin AJ, Huntington RW. Carcinoma arising in a retroperitoneal Müllerian cyst, with widespread metastasis during pregnancy. Am J Obstet Gynecol. 1965;91(2):210–6. 10.1016/0002-9378(65)90202-4 14258022

[2] Tykkä H, Koivuniemi A. Carcinoma arising in a mesenteric cyst. Am J Surg. 1975;129(6):709–11. 10.1016/0002-9610(75)90352-9 1130615

[3] Roth LM, Ehrlich CE. Mucinous cystadenocarcinoma of the retroperitoneum. Obstet Gynecol. 1977;49(4):486–8. 854250

[4] Fujii S, Konishi I, Okamura H, Mori T. Mucinous cystadenocarcinoma of the retroperitoneum: a light and electron microscopic study. Gynecol Oncol. 1986;24(1):103–12. 10.1016/0090-8258(86)90013-2 3699572

[5] Nelson H, Benjamin B, Alberty R. Primary retroperitoneal mucinous cystadenocarcinoma. Cancer. 1988;61(10):2117–21. 10.1002/1097-0142(19880515)61:10<2117::aid-cncr2820611031>3.0.co;2-x 3282642

[6] Chida T, Watanabe H, Motoyama T, Ajioka Y, Honma T, Kurosaki I, et al. A case of retroperitoneal mucinous cystadenocarcinoma. Gan No Rinsho. 1990;36(2):205–10. 2308212

[7] Seki H, Shiina M, Nishihara M, Kimura M, Kamura T, Sakai K, et al. Primary retroperitoneal mucinous cystadenocarcinoma: report of a case. Radiat Med. 8(5):164–7. 2075232

[8] Park U, Han KC, Chang HK, Huh MH. A primary mucinous cystoadenocarcinoma of the retroperitoneum. Gynecol Oncol. 1991;42(1):64–7. 10.1016/0090-8258(91)90232-t 1717354

[9] Jørgensen LJ, Vibits H (1991). Primary retroperitoneal mucinous cystadenocarcinoma. A case report and review of the literature. APMIS.

[10] Søndergaard, G. & Kaspersen, P. Ovarian and extraovarian mucinous tumors with solid mural nodules. Int J Gynecol Pathol 1991 10, 145–55. 10.1097/00004347-199104000-00003 1709621

[11] Gotoh K, Konaga E, Arata A, Takeuchi H, Mano SA (1992). Case of primary retroperitoneal mucinous cystadenocarcinoma. Acta Med Okayama.

[12] Tenti P, Carnevali L, Tateo S, Durola R (1994). Primary mucinous cystoadenocarcinoma of the retroperitoneum: two cases. Gynecol Oncol.

[13] Motoyama T, Chida T, Fujiwara T, Watanabe H. Mucinous cystic tumor of the retroperitoneum. A report of two cases. Acta Cyto. 1994;38:261–6. 8147222

[14] Carabias E, Garcia Muñoz H, Dihmes FP, López Pino MA, Ballestín C (1995). Primary mucinous cystadenocarcinoma of the retroperitoneum. Report of a case and literature review. Virchows Arch.

[15] Lee IW, Ching KC, Pang M, Ho TH (1996). Two cases of primary retroperitoneal mucinous cystadenocarcinoma. Gynecol Oncol.

[16] Dore R, et al. Primitive mucinous cystadenocarcinoma of the retroperitoneum. Case report and diagnostic considerations. Clin Imaging 1996;20:129–32. 10.1016/0899-7071(95)00004-6 8744823

[17] Uematsu T (2000). Ruptured retroperitoneal mucinous cystadenocarcinoma with synchronous gastric carcinoma and a long postoperative survival: case report. J Surg Oncol.

[18] Suzuki S, Mishina T, Ishizuka D, Fukase M, Matsubara YI (2001). Mucinous cystadenocarcinoma of the retroperitoneum: report of a case. Surg Today.

[19] Tangjitgamol S, et al. Retroperitoneal mucinous cystadenocarcinoma: a case report and review of literature. Int J Gynecol. Cancer 2002;12:403–8. 10.1046/j.1525-1438.2002.01120.x 12144691

[20] Kessler TM, Kessler W, Neuweiler J, Nachbur BH (2002). Treatment of a case of primary retroperitoneal mucinous cystadenocarcinoma: is adjuvant hysterectomy and bilateral salpingo-oophorectomy justified?. Am J Obstet Gynecol.

[21] Mikami M (2003). Retroperitoneal primary mucinous adenocarcinoma with a mural nodule of anaplastic tumor: a case report and literature review. Int J Gynecol Pathol.

[22] Song E-S (2005). Mucinous adenocarcinoma arising from one retroperitoneal mature cystic teratoma in a postmenopausal woman. J Obstet Gynaecol Res.

[23] Sonntag B (2005). Retroperitoneal mucinous adenocarcinoma occuring during pregnancy in a supernumerary ovary. J Obstet Gynaecol (Lahore).

[24] Thamboo TP, Sim R, Tan S-Y, Yap W-M (2006). Primary retroperitoneal mucinous cystadenocarcinoma in a male patient. J Clin Pathol.

[25] Fan YS, Thomas TMM, Ip PPC, Cheung ANY (2006). Osteoid-forming sarcoma-like mural nodule in a retroperitoneal mucinous cystadenocarcinoma. Histopathology.

[26] Law KS, Chang TM, Tung JN (2006). Fertility-sparing treatment of a primary retroperitoneal mucinous cystadenocarcinoma. BJOG.

[27] de León DC (2007). Primary retroperitoneal mucinous cystadenocarcinoma: report of two cases. World J Surg Oncol.

[28] Kashima K, Yahata T, Fujita K. Tanaka K. Primary retroperitoneal mucinous cystadenocarcinoma associated with pregnancy. Int J Gynecol Cancer. 2008;18:908–12. 10.1111/j.1525-1438.2007.01130.x 18028384

[29] Lee SA (2007). Primary retroperitoneal mucinous cystadenocarcinoma: a case report and review of the literature. Korean J Intern Med.

[30] Green JM, Bruner BC, Tang WW, Orihuela E. Retroperitoneal mucinous cystadenocarcinoma in a man: case report and review of the literature. Urol Oncol. 2007;25:53–5. 10.1016/j.urolonc.2006.02.015 17208139

[31] Tjalma WAA, Vaneerdeweg W (2007). Primary retroperitoneal mucinous cystadenocarcinomas are a distinct entity. Int J Gynecol Cancer.

[32] Moral González M, García-Blanch de Benito G, Sánchez Gil A, Díaz García GA, Cuberes Monserrat R (2008). Primary retroperitoneal mucinous cystadenocarcinoma. Cir Esp.

[33] Youssef C (2008). Primary retroperitoneal endometrial cystadenocarcinoma presenting as pelvic abscess on ultrasound. Ultrasound Obstet Gynecol.

[34] Roma AA, Malpica A (2009). Primary retroperitoneal mucinous tumors: a clinicopathologic study of 18 cases. Am J Surg Pathol.

[35] Hrora A (2009). Primary retroperitoneal mucinous cystadenocarcinoma in a male patient: a case report. Cases J.

[36] Dierickx I (2010). Primary retroperitoneal mucinous cystadenocarcinoma: a case report and review of the literature. Gynecol Obstet Investig.

[37] Jiang H, Jin K, You Q, Fang W, Xu N (2011). Retroperitoneal primary mucinous adenocarcinoma: a case report. Oncol Lett.

[38] Kanayama T (2012). Primary retroperitoneal mucinous cystadenocarcinoma with mural nodules: a case report and literature review. Int J Clin Oncol.

[39] Feng J, Liu H, Chen D (2013). Primary retroperitoneal mucinous cystadenocarcinoma in a male patient: a rare case report. Hippokratia.

[40] Hanhan HM (2014). Primary retroperitoneal mucinous cystadenocarcinoma during pregnancy. J Obstet Gynaecol.

[41] Shiau J-P, Wu C-T, Chin C-C, Chuang C-K (2013). Long-term survival after hand-assisted laparoscopic approach of primary retroperitoneal mucinous cystadenocarcinoma in male: case report and review of literature. Eur Surg.

[42] Kurita T, Nakajima K, Koi C, Matsuura Y, Hachisuga T (2014). Management of a primary retroperitoneal mucinous cystadenocarcinoma: case report. Eur J Gynaecol Oncol.

[43] Kamiyama H (2015). Report of a case: retroperitoneal mucinous cystadenocarcinoma with rapid progression. Int J Surg Case Rep.

[44] Cupp JS, Illeck J, Rahbar N, Rettenmaier MA, Goldstein BH. A rare case of primary retroperitoneal mucinous adenocarcinoma: a case report J Reprod Med. 2015;58:85–8. 23447927

[45] Dong B, Zhou H, Zhang J, Wang Y, Fu Y. Diagnosis and treatment of retroperitoneal bronchogenic cysts: a case report. Oncol Lett. 2014;7:2157–9. 10.3892/ol.2014.1974 PMC4049691 24932307

[46] Myriokefalitaki E, Luqman I, Potdar N, Brown L, Steward W, Moss EL. Primary retroperitoneal mucinous cystadenocarcinoma (PRMCa): a systematic review of the literature and meta-analysis. Arch Gynecol Obstet. 2016;293(4):709–20. 10.1007/s00404-015-3975-8 26681306

[47] Storch MP, Raghavan U. Mucinous cystadenocarcinoma of retroperitoneum. Conn Med. 1980;44(3):140–1. 7363617

[48] Pennell TC, Gusdon JP. Retroperitoneal mucinous cystadenoma. Am J Obstet Gynecol. 1989;160(5 Pt 1):1229–31. 10.1016/0002-9378(89)90201-9 2729400

[49] Abascal J, Ardaiz J, Gil P, Menéndez J, Barreiro JJ, Inchausti JL. [Primary retroperitoneal cyst (possible intestinal origin)]. Rev Esp Enferm Apar Dig . 1977 51(7):819–828. 594482

[50] Pearl ML, Valea F, Chumas J, Chalas E. Primary retroperitoneal mucinous cystadenocarcinoma of low malignant potential: a case report and literature review. Gynecol Oncol. 1996;61(1):150–2. 10.1006/gyno.1996.0115 8626105

